# NEW DIRECTIONS IN RESEARCH ON THE DYNAMICS OF PURPOSE IN LIFE AND COGNITIVE AGING

**DOI:** 10.1093/geroni/igad104.1024

**Published:** 2023-12-21

**Authors:** Angelina Sutin

## Abstract

Purpose in life is the feeling that one’s life is goal oriented and has direction. There is converging, replicated evidence that having a greater sense of purpose in life is associated with better cognitive aging outcomes, including less cognitive decline across middle and older adulthood and lower risk of incident dementia. Emerging evidence also suggests that cognitive function may likewise support purpose in life. This symposium highlights recent advances on the dynamics between purpose in life and cognition across varying time scales and populations. Kim will describe the association between purpose in life and the maintenance of better cognitive function across six years in older adulthood and moderators of this association. Nelson and Bergeman will describe how cognitive function supported purpose in life through the uncertainty of the Covid-19 pandemic and moderators of this association. Pfund and colleagues will report on the bidirectional associations between purpose and cognition in older adulthood across four longitudinal samples using a coordinated data analytic approach. Finally, Sutin and colleagues will report on an ecological momentary assessment study that found that in moments when participants felt more purposeful, they had faster processing speed. Collectively, these talks emphasize the dynamic and often bidirectional relation between purpose in life and cognitive function. This work will help stimulate research to better understand purpose and cognitive health and how to leverage purpose in life for healthier cognitive aging outcomes.

